# TFBSTools: an R/bioconductor package for transcription factor binding site analysis

**DOI:** 10.1093/bioinformatics/btw024

**Published:** 2016-01-21

**Authors:** Ge Tan, Boris Lenhard

**Affiliations:** Computational Regulatory Genomics, MRC Clinical Sciences Centre, Imperial College London, London W12 0NN, UK

## Abstract

**Summary**: The ability to efficiently investigate transcription factor binding sites (TFBSs) genome-wide is central to computational studies of gene regulation. *TFBSTools* is an R/Bioconductor package for the analysis and manipulation of TFBSs and their associated transcription factor profile matrices. *TFBStools* provides a toolkit for handling TFBS profile matrices, scanning sequences and alignments including whole genomes, and querying the JASPAR database. The functionality of the package can be easily extended to include advanced statistical analysis, data visualization and data integration.

**Availability and implementation**: The package is implemented in R and available under GPL-2 license from the Bioconductor website (http://bioconductor.org/packages/TFBSTools/).

**Contact:** ge.tan09@imperial.ac.uk

**Supplementary information:** Supplementary data are available at *Bioinformatics* online.

## 1 Introduction

Transcription factor binding sites (TFBSs) on DNA play a central role in gene regulation via their sequence-specific interaction with transcription factor (TF) proteins (Wasserman and Sandelin, 2004). Most individual TFBSs are 4–30 base-pairs (bp) wide, but are generally located in larger *cis*-regulatory regions of 50–200 bp. Analysis and identification of TFBSs is crucial for understanding the regulatory mechanisms of gene regulation.

At present, the TFBS analysis functionality in R/Bioconductor (Gentleman *et al.*, 2004) is limited and scattered across multiple packages. Here we introduce an R package *TFBSTools*, which provides a unified and efficiently implemented suite of TFBS analysis tools. The package provides a number of functions for manipulating TFBS profile matrices and searching DNA sequence and pairwise alignments using them. We have ported all of the functionality of our popular TFBS Perl modules (Lenhard and Wasserman, 2002), retaining the equivalent class structure where possible, and expanded the functionality to provide efficient genome-wide analysis of TFBSs. Our implementation is tightly integrated with the existing Bioconductor core packages, enabling high-performance sequence and interval manipulation. A database interface for JASPAR2014 (Mathelier *et al.*, 2014), JASPAR2016 (Mathelier *et al.*, 2015) and wrapper function for *de novo* motif discovery software are also provided.

## 2 Methods

### 2.1 S4 classes defined in TFBSTools

**Fig. 1.**
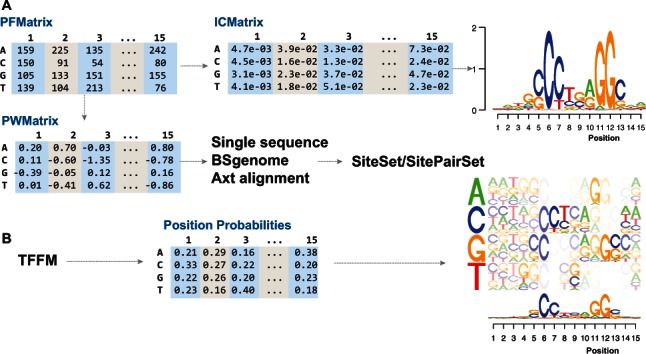
A common workflow and classes in TFBSTools. (**A**) *PFMatrix* can be converted into *PWMatrix*, *ICMatrix*. *ICMatrix* produces the sequence logos. *PWMatrix* scans the single sequence or alignment to produce *SiteSet* object that holds transcription factor binding sites. (**B**) *TFFM*: A virtual class for TFFM; *TFFMFirst* and *TFFMDetail* are derived from this virtual class. They can produce the position probabilities and the novel graphics representation of TFFM

To provide easy data storage, manipulation and exchange, we created several novel S4 classes (Fig. 1), and also defined an aggregate version of each class (e.g. *PFMatrixList*) to help manipulate sets of the corresponding objects. The design of these classes corresponds to classes in TFBS Perl modules, while remaining extensible in an object-oriented manner, adding new functionality and taking advantage of functional programming capabilities of R.

### 2.2 Operations with TFBS matrix profiles

To characterize the binding preference of a TF, the aligned sequences bound by the TF are aggregated into a position frequency matrix (PFM). From this matrix, another two matrices can be derived: position weight matrix (PWM, the most commonly used kind of position-specific scoring matrix) and information content matrix (ICM). PWM is a matrix of positional log-likelihoods normally used for sequence scanning and scoring against the motif, while ICM is mostly used in motif visualization, e.g. for drawing sequence logos which can be easily done by the package *seqLogo* (Fig. 1A). As a novel feature, in addition to matrix profiles, TFBSTools also supports the manipulation of transcription factor flexible model (TFFM) profiles (Mathelier and Wasserman, 2013), which capture the dinucleotide dependence (Fig. 1B).

*TFBSTools* provides methods to perform the conversion between different types of matrices, providing a range of options and customizations. The highlights include: (i) a default pseudocount of 0.8 (Nishida *et al.*, 2009) is used to eliminate the small or zero counts before log transformation, although a different pseudocount, or pseudocount function, for each column is possible; (ii) Schneider correction for ICM is available; (iii) Unequal background nucleotide frequencies can also be specified.

*TFBSTools* provides tools for comparing pairs of PFMs, or a PFM with IUPAC strings, using a modified Needleman–Wunsch algorithm (Sandelin *et al.*, 2003). Quantification of the similarity between PFMs is commonly used for comparing a newly discovered matrix with existing matrices in the motif database, such as JASPAR, to determine whether the motif is related to known annotated motifs.

The similarity between two PWMs can be quantified using several metrics (e.g. normalized Euclidian distance, Pearson correlation coefficient and Kullback–Leibler divergence). In addition, *TFBSTools* also allows random profile generation by: (i) sampling the posterior distribution of Dirichlet multinomial mixture models trained on all available JASPAR matrices; (ii) permutation of columns from selected PFMs. The availability of random matrices with the same statistical properties as selected profiles is particularly useful for computational/simulation studies, such as matrix-matrix comparison.

### 2.3 Sequence/alignment scanning with PWM profiles

*TFBSTools* includes facilities for screening potential TFBSs present in a DNA sequence (searchSeq), or conserved in a pairwise alignment.

When a pairwise alignment is available, it can be used to combine the TFBSs prediction with phylogenetic footprinting, which can in many cases reduce the false discovery rate whilst retaining a sufficient level of sensitivity (Wasserman and Sandelin, 2004). Alternatively, it can be used in combination with other data (e.g. ChIP-seq) to study the cross-species conservation properties of TF binding.

For genome-wide phylogenetic footprinting, *TFBSTools* can accept two *BSgenome* objects, and a chain file for *liftover* from one genome to another (searchPairBSgenome) or a novel S4 class *Axt* from our *CNEr* package (available from the Bioconductor website) for representing the axt alignments (searchAln). It can take up to 50 CPU hours to run searchAln on human–mouse pairwise alignment with the possibility of parallel computation, while searchSeq or searchPairBSgenome only needs several minutes. The computationally predicted putative TFBSs can be returned in GFF format or *GRanges* for downstream analysis.

### 2.4 JASPAR database interface

Since the release of JASPAR2014 (Mathelier *et al.*, 2014), we have provided Bioconductor data packages, *JASPAR2014* and *JASPAR2016*, holding the profile matrices and associated metadata. To accompany the use of this data package for TFBS analysis, TFBSTools provides functions to enable efficient database querying and manipulation.

### 2.5 Use of *de novo* motif discovery software

*TFBSTools* provides wrapper functions for *de novo* motif discovery softwares and seamlessly integrates the results back into R objects. Currently, support for *MEME* is implemented and reported motifs are stored in *MotifSet* object.

## 3 Conclusions and further information

The Bioconductor *TFBSTools* package provides a full suite of TFBS analysis tools. The package allows the efficient and reproducible identification and analysis of TFBSs. In combination with other functionality in Bioconductor, it provides a powerful way to analyze TF binding motifs on genome-wide scale. Further development will include an efficient implementation of scanning sequence/alignment with TFFM. A tutorial and additional use cases are available at Bioconductor website.

## Supplementary Material

Supplementary Data
