# Supplementary material for: TFBSTools: an R/bioconductor package for transcription factor binding site analysis
Source: Bioinformatics. 2016 Jan 21;32(10):1555–6. doi: 10.1093/bioinformatics/btw024 (PMC4866524; doi:10.1093/bioinformatics/btw024)
Supplement: Supplementary Data [file supp_32_10_1555__index.html]

TFBSTools: an R/bioconductor package for transcription factor binding site analysis — TFBSTools: an R/bioconductor package for transcription factor binding site analysis — Supplementary Data 

# TFBSTools: an R/bioconductor package for transcription factor binding site analysis

## Supplementary Data

files

- Supplementary Data - pdf file
