# Supplementary material for: TFBSTools: an R/bioconductor package for transcription factor binding site analysis
Source: Bioinformatics. 2016 Jan 21;32(10):1555–6. doi: 10.1093/bioinformatics/btw024 (PMC4866524; doi:10.1093/bioinformatics/btw024)
Supplement: Supplementary Data [file supp_btw024_btw024_Tan_TFBSTools_BioC_Vignette.pdf]

The *TFBSTools* package overview

Ge Tan *

Edited: Sep 2015; Compiled: November 9, 2015

Contents

1 Introduction **2**

2 S4 classes in TFBSTools **2**
- 2.1 XMatrix and its subclasses . . . 2
- 2.2 XMatrixList and its subclasses . . . 4
- 2.3 SiteSet, SiteSetList, SitePairSet and SitePairSetList . . . 5
- 2.4 MotifSet . . . 5
- 2.5 TFFM and its subclasses . . . 5

3 Database interfaces for JASPAR2014 database **5**
- 3.1 Search JASPAR2014 database . . . 6
- 3.2 Store, delete and initialize JASPAR2014 database . . . 6

4 PFM, PWM and ICM methods **7**
- 4.1 PFM to PWM . . . 7
- 4.2 PFM to ICM . . . 8
- 4.3 Align PFM to a custom matrix or IUPAC string . . . 10
- 4.4 PWM similarity . . . 11
- 4.5 Dynamic random profile generation . . . 11

5 TFFM methods **13**
- 5.1 The graphical representation of TFFM . . . 13

6 Scan sequence and alignments with PWM pattern **15**
- 6.1 searchSeq . . . 15
- 6.2 searchAln . . . 17
- 6.3 searchPairBSgenome . . . 20

7 Use de novo motif discovery software **20**
- 7.1 *MEME* . . . 20

8 Session info **20**

*ge.tan09@imperial.ac.uk

1 Introduction

Eukaryotic regulatory regions are characterized based a set of discovered transcription factor binding sites (TFBSs), which can be represented as sequence patterns with various degree of degeneracy.

This *TFBSTools* package is designed to be a compuational framework for TFBSs analysis. Based on the famous perl module TFBS [1], we extended the class definitions and enhanced implementations in an interactive environment. So far this package contains a set of integrated *R* `S4` style classes, tools, JASPAR database interface functions. Most approaches can be described in three sequential phases. First, a pattern is generated for a set of target sequences known to be bound by a specific transcription factor. Second, a set of DNA sequences are analyzed to determine the locations of sequences consistent with the described binding pattern. Finally, in advanced cases, predictive statistical models of regulatory regions are constructed based on mutiple occurrences of the detected patterns.

Since JASPAR2016, the next generation of transcription factor binding site, TFFM [2], was introduced into JASPAR for the first time. Now *TFBSTools* also supports the manipulation of TFFM. TFFM is based on hidden Markov Model (HMM). The biggest advantage of TFFM over basic PWM is that it can model position interdependence within TFBSs and variable motif length. A novel graphical representation of the TFFM motifs that captures the position interdependence is also introduced. For more details regarding TFFM, please refer to http://cisreg.cmmt.ubc.ca/TFFM/doc/.

TFBSTools aims to support all these functionalities in the environment *R*, except the external motif finding software, such as *MEME* [3].

2 `S4` classes in TFBSTools

The package is built around a number of `S4` class of which the *XMatrix*, *SiteSet* classes are the most important. The section will briefly explain most of them defined in *TFBSTools*.

2.1 XMatrix and its subclasses

XMatrix is a virtual class, which means no concrete objects can be created directly from it. The subclass *PFMatrix* is designed to store all the relevant information for one raw position frequency matrix (PFM). This object is compatible with one record from JASPAR database. *PWMatrix* is used to store a position weight matrix (PWM). Compared with *PFMatrix*, it has one extra slot `pseudocounts`. *ICMatrix* is used to store a information content matrix (ICM). Compared with *PWMatrix*, it has one extra slot `schneider`.

The following examples demonstrate the creation of *PFMatrix*, the conversions between these matrices and some assocated methods defined for these classes.

```
## PFMatrix construction; Not all of the slots need to be initialised.
pfm = PFMatrix(ID="MA0004.1", name="Arnt", matrixClass="Zipper-Type", strand="+",
        bg=c(A=0.25, C=0.25, G=0.25, T=0.25),
        tags=list(family="Helix-Loop-Helix", species="10090",
                  tax_group="vertebrates",medline="7592839", type="SELEX",
                  ACC="P53762", pazar_tf_id="TF0000003",
                  TFBSshape_ID="11", TFencyclopedia_ID="580"),
        profileMatrix=matrix(c(4L,  19L, 0L,  0L,  0L,  0L,
                               16L, 0L,  20L, 0L,  0L,  0L,
                               0L,  1L,  0L,  20L, 0L,  20L,
                               0L,  0L,  0L,  0L,  20L, 0L),
```

```
                             byrow=TRUE, nrow=4,
                             dimnames=list(c("A", "C", "G", "T")))
        )

pfm

## An object of class PFMatrix
## ID: MA0004.1
## Name: Arnt
## Matrix Class: Zipper-Type
## strand: +
## Tags:
## $family
## [1] "Helix-Loop-Helix"
##
## $species
## [1] "10090"
##
## $tax_group
## [1] "vertebrates"
##
## $medline
## [1] "7592839"
##
## $type
## [1] "SELEX"
##
## $ACC
## [1] "P53762"
##
## $pazar_tf_id
## [1] "TF0000003"
##
## $TFBSshape_ID
## [1] "11"
##
## $TFencyclopedia_ID
## [1] "580"
##
## Background:
##    A    C    G    T
## 0.25 0.25 0.25 0.25
## Matrix:
##   [,1] [,2] [,3] [,4] [,5] [,6]
## A    4   19    0    0    0    0
## C   16    0   20    0    0    0
## G    0    1    0   20    0   20
## T    0    0    0    0   20    0

## coerced to matrix
as.matrix(pfm)
```

```
##   [,1] [,2] [,3] [,4] [,5] [,6]
## A    4   19    0    0    0    0
## C   16    0   20    0    0    0
## G    0    1    0   20    0   20
## T    0    0    0    0   20    0

## access the slots of pfm
ID(pfm)

## [1] "MA0004.1"

name(pfm)

## [1] "Arnt"

Matrix(pfm)

##   [,1] [,2] [,3] [,4] [,5] [,6]
## A    4   19    0    0    0    0
## C   16    0   20    0    0    0
## G    0    1    0   20    0   20
## T    0    0    0    0   20    0

ncol(pfm)

## [1] 6

length(pfm)

## [1] 6

## convert a FPM to PWM, ICM
pwm = toPWM(pfm, type="log2probratio", pseudocounts=0.8,
            bg=c(A=0.25, C=0.25, G=0.25, T=0.25))

icm = toICM(pfm, pseudocounts=sqrt(rowSums(pfm)[1]), schneider=FALSE,
            bg=c(A=0.25, C=0.25, G=0.25, T=0.25))

## get the reverse complment matrix with all the same information except the strand.
pwmRevComp <- reverseComplement(pwm)
```

2.2 XMatrixList and its subclasses

XMatrixList is used to store a set of *XMatrix* objects. Basically it is a SimpleList for easy manipulation the whole set of *XMatrix*. The concrete objects can be *PFMatrix*, *PWMatrix* and *ICMatrix*.

```
pfm2 = pfm
pfmList = PFMatrixList(pfm1=pfm, pfm2=pfm2, use.names=TRUE)
pfmList

## PFMatrixList of length 2
## names(2): pfm1 pfm2

names(pfmList)

## [1] "pfm1" "pfm2"
```

2.3 SiteSet, SiteSetList, SitePairSet and SitePairSetList

The *SiteSet* class is a container for storing a set of putative transcription factor binding sites on a nucleotide sequence (start, end, strand, score, pattern as a *PWMatrix*, etc.) from scaning a nucleotide sequence with the corresponding *PWMatrix*. Similarly, *SiteSetList* stores a set of *SiteSet* objects.

For holding the results returned from a pairwise alignment scaaning, *SitePairSet* and *SitePairSetList* are provided.

More detailed examples of using these classes will be given in Section 6;

2.4 MotifSet

This *MotifSet* class is used to store the generated motifs from *de novo* motif discovery software, such as *MEME* [3].

2.5 TFFM and its subclasses

TFMM is a virtual class and two classes *TFFMFirst* and *TFFMDetail* are derived from this virtual class. Compared with *PFMatrix* class, *TFFM* has two extra slots that store the emission distribution parameters and transition probabilities. *TFFMFirst* class stands for the first-order TFFMs, while *TFFMDetail* stands for the more detailed and descriptive TFFMs.

Although we provide the constructor functions for *TFFM* class, the *TFFM* object is usually generated from reading a XML file from the Python module *TFFM*.

```
xmlFirst <- file.path(system.file("extdata", package="TFBSTools"),
                     "tffm_first_order.xml")
tffmFirst <- readXMLTFFM(xmlFirst, type="First")
tffm <- getPosProb(tffmFirst)

xmlDetail <- file.path(system.file("extdata", package="TFBSTools"),
                      "tffm_detailed.xml")
tffmDetail <- readXMLTFFM(xmlDetail, type="Detail")
getPosProb(tffmDetail)

##        1      2      3      4       5        6       7       8       9     10       11
## A 0.2115 0.2839 0.1638 0.1872 0.03682 0.005747 0.01884 0.04937 0.07792 0.4653 0.128593
## C 0.3348 0.2854 0.2442 0.1841 0.41600 0.935778 0.77794 0.33508 0.43249 0.1519 0.078589
## G 0.2279 0.2737 0.2134 0.4209 0.49586 0.055218 0.07339 0.16227 0.40736 0.3457 0.786246
## T 0.2259 0.1570 0.3787 0.2079 0.05132 0.003256 0.12982 0.45328 0.08222 0.0371 0.006573
##         12      13     14     15
## A 0.003277 0.04504 0.2113 0.3662
## C 0.067743 0.51743 0.4023 0.2161
## G 0.924718 0.40149 0.1895 0.2429
## T 0.004262 0.03604 0.1969 0.1747
```

3 Database interfaces for JASPAR2014 database

This section will demonstrate how to operate on the JASPAR 2014 database. JASPAR is a collection of transcription factor DNA-binding preferences, modeled as matrices. These can be converted into PWMs,

used for scanning genomic sequences. JASPAR is the only database with this scope where the data can be used with no restrictions (open-source). A *Bioconductor* experiment data package *JASPAR2014* is provided with each release of JASPAR.

3.1 Search JASPAR2014 database

This search function fetches matrix data for all matrices in the database matching criteria defined by the named arguments and returns a PFMatrixList object. For more search criterias, please see the help page for `getMatrixSet`.

```
suppressMessages(library(JASPAR2014))
opts = list()
opts[["species"]] = 9606
opts[["name"]] = "RUNX1"
#opts[["class"]] = "Ig-fold"
opts[["type"]] = "SELEX"
opts[["all_versions"]] = TRUE
PFMatrixList = getMatrixSet(JASPAR2014, opts)
PFMatrixList

## PFMatrixList of length 1
## names(1): MA0002.1

opts2 = list()
opts2[["type"]] = "SELEX"
PFMatrixList2 = getMatrixSet(JASPAR2014, opts2)
PFMatrixList2

## PFMatrixList of length 111
## names(111): MA0004.1 MA0006.1 MA0008.1 MA0009.1 ... MA0587.1 MA0588.1 MA0589.1 MA0590.1
```

3.2 Store, delete and initialize JASPAR2014 database

We also provide some functions to initialize an empty JASPAR2014 style database, store new *PFMatrix* or *PFMatrixList* into it, or delete some records based on ID. The backend of the database is *SQLite*.

```
db = "myMatrixDb.sqlite"
initializeJASPARDB(db, version="2014")

## [1] "Success"

data("MA0043")
storeMatrix(db, MA0043)

## [1] "Success"

deleteMatrixHavingID(db,"MA0043.1")
file.remove(db)

## [1] TRUE
```

4 PFM, PWM and ICM methods

This section will give an introduction of matrix operations, including conversion from PFM to PWM and ICM, profile matrices comparison, dynamic random profile generation.

4.1 PFM to PWM

The method toPWM can convert PFM to PWM [4]. Optional parameters include type, pseudocounts, bg. The implementation in this package is a bit different from that in *Biostrings*.

First of all, toPWM allows the input matrix to have different column sums, which means the count matrix can have an unequal number of sequences contributing to each column. This scenario is rare, but exists in JASPAR SELEX data.

Second, we can specify customized pseudocounts. pseudocounts is necessary for correcting the small number of counts or eliminating the zero values before log transformation. In TFBS perl module, the square root of the number of sequences contributing to each column. However, it has been shown to too harsh [5]. Hence, a default value of 0.8 is used. Of course, it can be changed to other customized value or even different values for each column.

```
pwm = toPWM(pfm, pseudocounts=0.8)
pwm

## An object of class PWMatrix
## ID: MA0004.1
## Name: Arnt
## Matrix Class: Zipper-Type
## strand: +
## Pseudocounts: 0.8
## Tags:
## $family
## [1] "Helix-Loop-Helix"
##
## $species
## [1] "10090"
##
## $tax_group
## [1] "vertebrates"
##
## $medline
## [1] "7592839"
##
## $type
## [1] "SELEX"
##
## $ACC
## [1] "P53762"
##
## $pazar_tf_id
## [1] "TF0000003"
##
## $TFBSshape_ID
```

```
## [1] "11"
##
## $TFencyclopedia_ID
## [1] "580"
##
## Background:
##    A    C    G    T
## 0.25 0.25 0.25 0.25
## Matrix:
##       [,1]   [,2]   [,3]   [,4]   [,5]   [,6]
## A -0.3081  1.885 -4.700 -4.700 -4.700 -4.700
## C  1.6394 -4.700  1.958 -4.700 -4.700 -4.700
## G -4.7004 -2.115 -4.700  1.958 -4.700  1.958
## T -4.7004 -4.700 -4.700 -4.700  1.958 -4.700
```

4.2 PFM to ICM

The method `toICM` can convert PFM to ICM [6]. Besides the similar `pseudocounts`, `bg`, you can also choose to do the `schneider` correction.

The information content matrix has a column sum between 0 (no base preference) and 2 (only 1 base used). Usually this information is used to plot sequence log.

How a PFM is converted to ICM: we have the PFM matrix x, base backrgound frequency bg, $pseudocounts$ for correction.

$$Z[j] = \sum_{i=1}^{4} x[i,j] \tag{1}$$

$$p[i,j] = \frac{(x[i,j] + bg[i] \times pseudocounts[j])}{(Z[j] + \sum_i bg[i] \times pseudocounts[j]} \tag{2}$$

$$D[j] = \log_2 4 + \sum_{i=1}^{4} p[i,j] * \log p[i,j] \tag{3}$$

$$ICM[i,j] = p[i,j] \times D[j] \tag{4}$$

```
icm = toICM(pfm, pseudocounts=0.8, schneider=TRUE)
icm

## An object of class ICMatrix
## ID: MA0004.1
## Name: Arnt
## Matrix Class: Zipper-Type
## strand: +
## Pseudocounts: 0.8
## Schneider correction: TRUE
## Tags:
## $family
## [1] "Helix-Loop-Helix"
##
## $species
```

```
## [1] "10090"
##
## $tax_group
## [1] "vertebrates"
##
## $medline
## [1] "7592839"
##
## $type
## [1] "SELEX"
##
## $ACC
## [1] "P53762"
##
## $pazar_tf_id
## [1] "TF0000003"
##
## $TFBSshape_ID
## [1] "11"
##
## $TFencyclopedia_ID
## [1] "580"
##
## Background:
##    A    C    G    T
## 0.25 0.25 0.25 0.25
## Matrix:
##          [,1]    [,2]    [,3]    [,4]    [,5]    [,6]
## A 0.203986 1.30440 0.01588 0.01588 0.01588 0.01588
## C 0.786802 0.01359 1.60404 0.01588 0.01588 0.01588
## G 0.009714 0.08152 0.01588 1.60404 0.01588 1.60404
## T 0.009714 0.01359 0.01588 0.01588 1.60404 0.01588
```

To plot the sequence logo, we use the package *seqlogo*. In sequence logo, each position gives the information content obtained for each nucleotide. The higher of the letter corresponding to a nucleotide, the larger information content and higher probability of getting that nucleotide at that position.

```
seqLogo(icm)
```

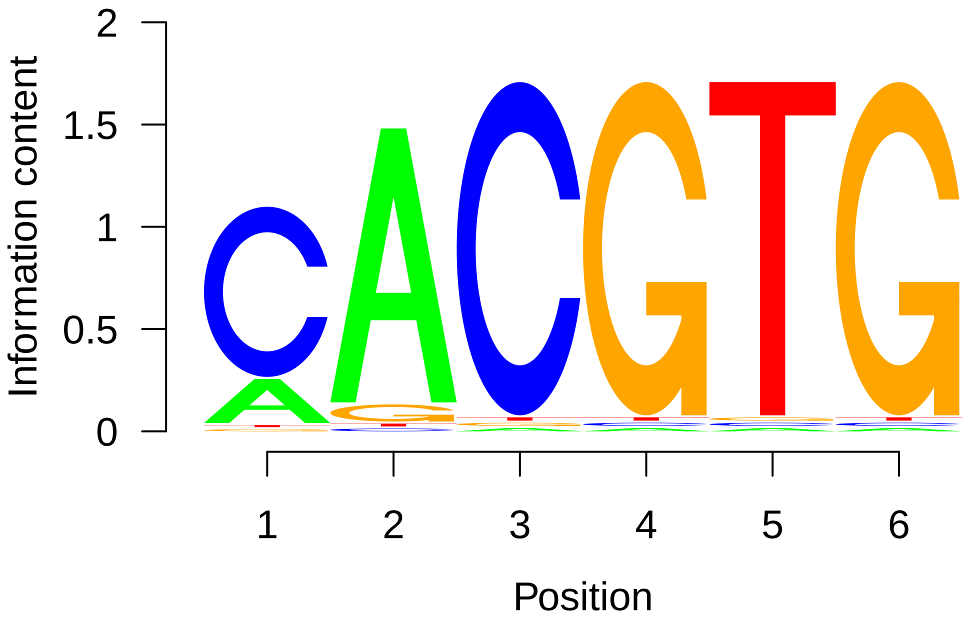

4.3 Align PFM to a custom matrix or IUPAC string

In some cases, it is beneficial to assess similarity of existing profile matrices, such as JASPAR, to a newly discovered matrix (as with using BLAST for sequence data comparison when using Genbank).

TFBSTools provides tools for comparing pairs of PFMs, or a PFM with IUPAC string, using a modified Needleman-Wunsch algorithm [7].

```
## one to one comparison
data(MA0003.2)
data(MA0004.1)
pfmSubject = MA0003.2
pfmQuery =  MA0004.1
PFMSimilarity(pfmSubject, pfmQuery)

##    score relScore
##    8.104   27.012

## one to several comparsion
PFMSimilarity(pfmList, pfmQuery)

## $pfm1
##    score relScore
##       12      100
##
## $pfm2
##    score relScore
##       12      100

## align IUPAC string
IUPACString = "ACGTMRWSYKVHDBN"
```

```r
PFMSimilarity(pfmList, IUPACString)

## $pfm1
##    score relScore
##    8.815   29.383
##
## $pfm2
##    score relScore
##    8.815   29.383
```

4.4 PWM similarity

To measure the similarity of two PWM matrix in three measurements: “normalised Euclidean distance”, “Pearson correlation” and “Kullback Leibler divergence” [8]. Given two PWMs, P^1 and P^2, where l is the length. $P_{i,b}$ is the values in column i with base b. The normalised Euclidean distance is computed in

$$D(a,b) = \frac{1}{\sqrt{2}l} \cdot \sum_{i=1}^{l} \sqrt{\sum_{b \in \{A,C,G,T\}} (P_{i,b}^1 - P_{i,b}^2)^2} \quad (5)$$

This distance is between 0 (perfect identity) and 1 (complete dis-similarity).

The pearson correlation coefficient is computed in

$$r(P^1,P^2) = \frac{1}{l} \cdot \sum_{i=1}^{l} \frac{\sum_{b \in \{A,C,G,T\}} (P_{i,b}^1 - 0.25)(P_{i,b}^2 - 0.25)}{\sqrt{\sum_{b \in \{A,C,G,T\}} (P_{i,b}^1 - 0.25)^2 \cdot \sum_{b \in \{A,C,G,T\}} (P_{i,b}^2 - 0.25)^2}}. \quad (6)$$

The Kullback-Leibler divergence is computed in

$$KL(P^1,P^2) = \frac{1}{2l} \cdot \sum_{i=1}^{l} \sum_{b \in \{A,C,G,T\}} (P_{i,b}^1 \log \frac{P_{i,b}^1}{P_{i,b}^2} + P_{i,b}^2 \log \frac{P_{i,b}^2}{P_{i,b}^1}). \quad (7)$$

```r
  data(MA0003.2)
  data(MA0004.1)
  pwm1 = toPWM(MA0003.2, type="prob")
  pwm2 = toPWM(MA0004.1, type="prob")
  PWMSimilarity(pwm1, pwm2, method="Euclidean")

## [1] 0.5135

  PWMSimilarity(pwm1, pwm2, method="Pearson")

## [1] 0.2829

  PWMSimilarity(pwm1, pwm2, method="KL")

## [1] 2.386
```

4.5 Dynamic random profile generation

In this section, we will demonstrate the capability of random profile matrices generation with matrix permutation and probabilitis sampling. In many computational/simulation studies, it is particularly desired

to have a set of random matrices. Some cases includes the estimation of distance between putative TFBS and transcription start site, the evaluation of comparison between matrices [9]. These random matrices are expected to have same statistical properties with the selcted profiles, such as nucleotide content or information content.

The permutation method is relatively easy. It simply shuffles the columns either constrainted in each matrix, or columns almong all selected matrices. The probabilistic sampling is more complicated and can be done in two steps:

1. A Dirichlet multinomial mixture model is trained on all available matrices in JASPAR.
2. Random columns are sampled from the posterior distribution of the trained Dirichlet model based on selected profiles.

```
## Matrice permutation
permuteMatrix(pfmQuery)
```

```
## An object of class PFMatrix
## ID: MA0004.1
## Name: Arnt
## Matrix Class: Zipper-Type
## strand: +
## Tags:
## $comment
## [1] "-"
##
## $family
## [1] "Helix-Loop-Helix"
##
## $medline
## [1] "7592839"
##
## $pazar_tf_id
## [1] "TF0000003"
##
## $tax_group
## [1] "vertebrates"
##
## $tfbs_shape_id
## [1] "11"
##
## $tfe_id
## [1] "580"
##
## $type
## [1] "SELEX"
##
## $collection
## [1] "CORE"
##
## $species
## [1] "10090"
##
## $acc
```

```
## [1] "P53762"
##
## Background:
##    A    C    G    T
## 0.25 0.25 0.25 0.25
## Matrix:
##   [,1] [,2] [,3] [,4] [,5] [,6]
## A    0    0    0    0   19    4
## C    0    0    0   20    0   16
## G   20    0   20    0    1    0
## T    0   20    0    0    0    0
```

```
permuteMatrix(pfmList, type="intra")
```

```
## PFMatrixList of length 2
## names(2): pfm1 pfm2
```

```
permuteMatrix(pfmList, type="inter")
```

```
## PFMatrixList of length 2
## names(2): pfm1 pfm2
```

```
## Dirichlet model training
data(MA0003.2)
data(MA0004.1)
pfmList <- PFMatrixList(pfm1=MA0003.2, pfm2=MA0004.1, use.names=TRUE)
dmmParameters <- dmmEM(pfmList, K=6, alg="C")
## Matrice sampling from trained Dirichlet model
pwmSampled <- rPWMDmm(MA0003.2, dmmParameters$alpha0, dmmParameters$pmix,
                      N=1, W=6)
```

5 TFFM methods

5.1 The graphical representation of TFFM

Basic PWMs can be graphically represented by the sequence logos shown above. A novel graphical representation of TFFM is requied for taking the dinucleotide dependence into account.

For the upper part of the sequence logo, we represent the nucleotide probabilities at position p for each possible nucleotide at position $p-1$. Hence, each column represents a position within a TFBS and each row the nucleotide probabilities found at that position. Each row assumes a specific nucleotide has been emitted by the previous hidden state. The intersection between a column corresponding to position p and row corresponding to nucleotide n gives the probabilities of getting each nucleotide at position p if n has been seen at position $p-1$. The opacity to represent the sequence logo is proportional to the probablity of possible row to be used by the TFFM.

```
## sequence logo for First-order TFFM
seqLogo(tffmFirst)
```

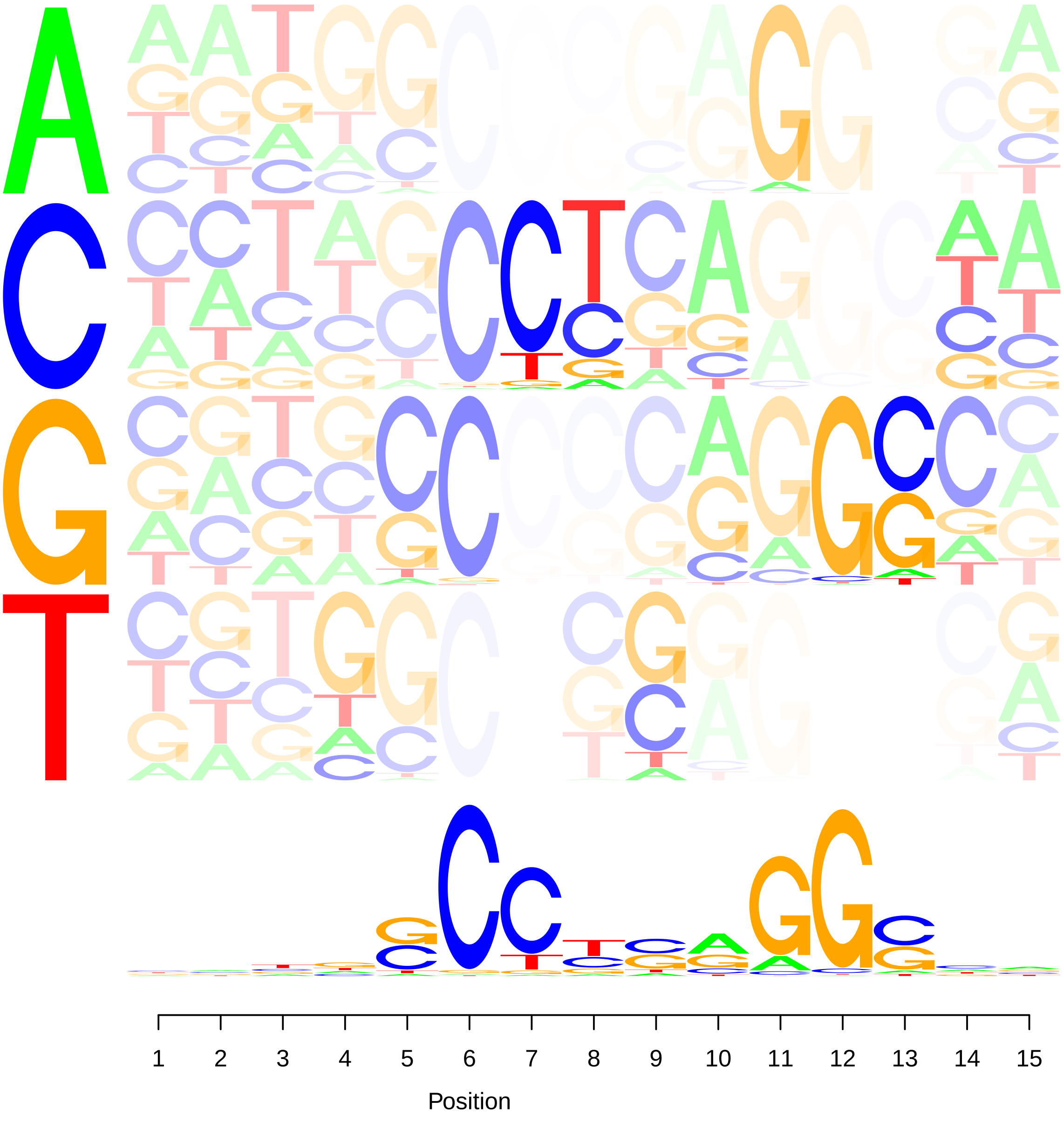


```
## sequence logo for detailed TFFM
seqLogo(tffmDetail)
```

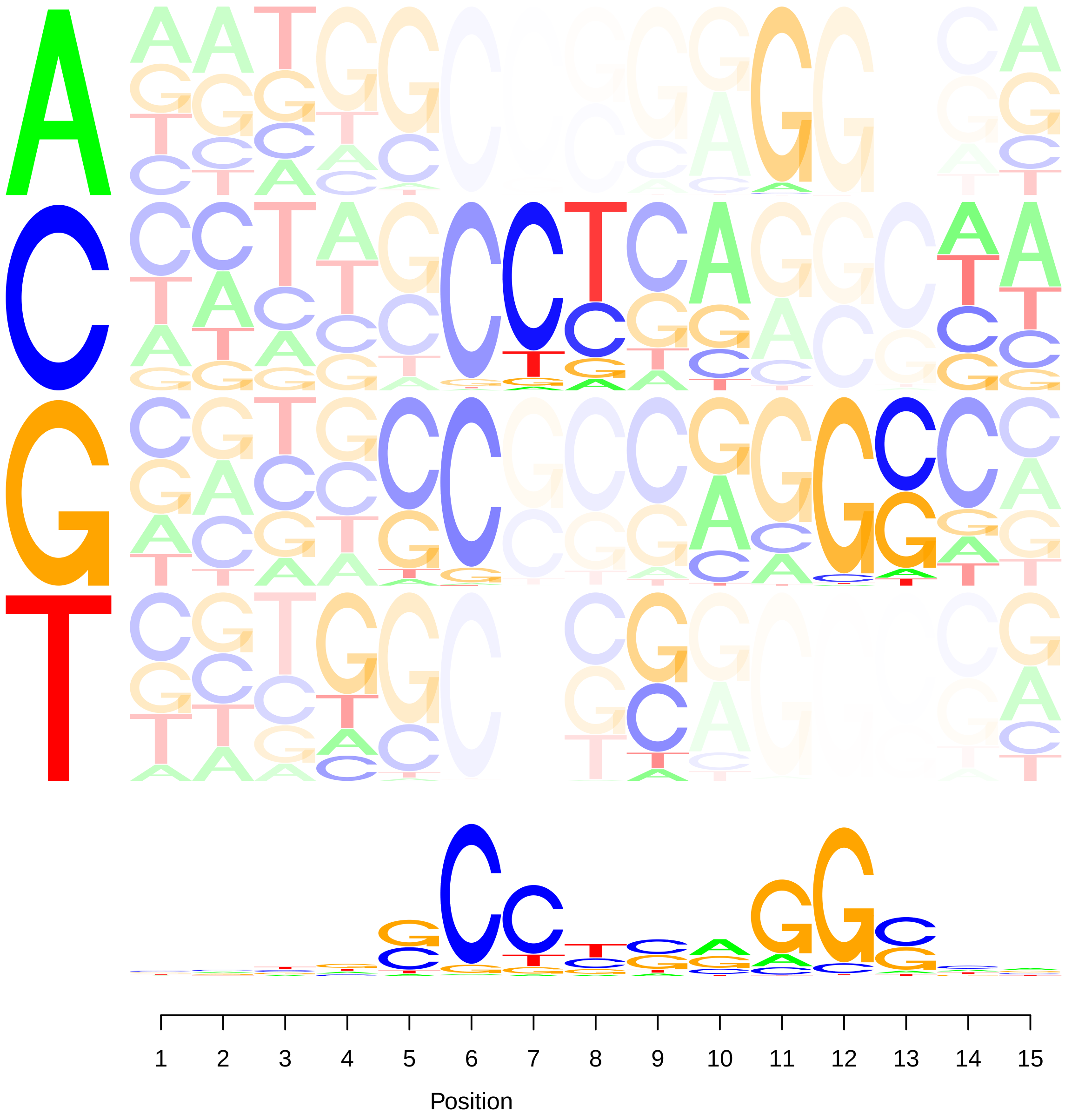

6 Scan sequence and alignments with PWM pattern

6.1 searchSeq

`searchSeq` scans a nucleotide sequence with the pattern represented in the PWM. The strand argument controls which strand of the sequence will be searched. When it is "*", both strands will be scanned.

A *siteset* object will be returned which can be exported into GFF3 or GFF2 format. Empirical p-values for the match scores can be calculated by an exact method from *TFMPvalue* or the distribution of sampled scores.

```
library(Biostrings)
data(MA0003.2)
data(MA0004.1)
```

```
pwmList = PWMatrixList(MA0003.2=toPWM(MA0003.2), MA0004.1=toPWM(MA0004.1),
                       use.names=TRUE)
subject = DNAString("GAATTCTCTCTTGTTGTAGTCTCTTGACAAAATG")
siteset = searchSeq(pwm, subject, seqname="seq1", min.score="60%", strand="*")

sitesetList = searchSeq(pwmList, subject, seqname="seq1",
                        min.score="60%", strand="*")


## generate gff2 or gff3 style output
head(writeGFF3(siteset))

##   seqname source feature start end  score strand frame
## 1    seq1   TFBS    TFBS     8  13 -1.888      +     .
## 2    seq1   TFBS    TFBS    21  26 -1.888      +     .
## 3    seq1   TFBS    TFBS    29  34 -3.909      +     .
## 4    seq1   TFBS    TFBS     8  13 -1.961      -     .
## 5    seq1   TFBS    TFBS    10  15 -3.909      -     .
## 6    seq1   TFBS    TFBS    21  26 -1.961      -     .
##                                 attributes
## 1 TF=Arnt;class=Zipper-Type;sequence=CTCTTG
## 2 TF=Arnt;class=Zipper-Type;sequence=CTCTTG
## 3 TF=Arnt;class=Zipper-Type;sequence=AAAATG
## 4 TF=Arnt;class=Zipper-Type;sequence=CAAGAG
## 5 TF=Arnt;class=Zipper-Type;sequence=AACAAG
## 6 TF=Arnt;class=Zipper-Type;sequence=CAAGAG

head(writeGFF3(sitesetList))

##            seqname source feature start end   score strand frame
## MA0003.2      seq1   TFBS    TFBS    18  32 -16.438      -     .
## MA0004.1.1    seq1   TFBS    TFBS     8  13  -1.888      +     .
## MA0004.1.2    seq1   TFBS    TFBS    21  26  -1.888      +     .
## MA0004.1.3    seq1   TFBS    TFBS    29  34  -3.909      +     .
## MA0004.1.4    seq1   TFBS    TFBS     8  13  -1.961      -     .
## MA0004.1.5    seq1   TFBS    TFBS    10  15  -3.909      -     .
##                                                  attributes
## MA0003.2   TF=TFAP2A;class=Zipper-Type;sequence=TTTTGTCAAGAGACT
## MA0004.1.1            TF=Arnt;class=Zipper-Type;sequence=CTCTTG
## MA0004.1.2            TF=Arnt;class=Zipper-Type;sequence=CTCTTG
## MA0004.1.3            TF=Arnt;class=Zipper-Type;sequence=AAAATG
## MA0004.1.4            TF=Arnt;class=Zipper-Type;sequence=CAAGAG
## MA0004.1.5            TF=Arnt;class=Zipper-Type;sequence=AACAAG

head(writeGFF2(siteset))

##   seqname source feature start end  score strand frame
## 1    seq1   TFBS    TFBS     8  13 -1.888      +     .
## 2    seq1   TFBS    TFBS    21  26 -1.888      +     .
## 3    seq1   TFBS    TFBS    29  34 -3.909      +     .
## 4    seq1   TFBS    TFBS     8  13 -1.961      -     .
## 5    seq1   TFBS    TFBS    10  15 -3.909      -     .
## 6    seq1   TFBS    TFBS    21  26 -1.961      -     .
```

```
##                                                  attributes
## 1 TF "Arnt"; class "Zipper-Type"; sequence "CTCTTG"
## 2 TF "Arnt"; class "Zipper-Type"; sequence "CTCTTG"
## 3 TF "Arnt"; class "Zipper-Type"; sequence "AAAATG"
## 4 TF "Arnt"; class "Zipper-Type"; sequence "CAAGAG"
## 5 TF "Arnt"; class "Zipper-Type"; sequence "AACAAG"
## 6 TF "Arnt"; class "Zipper-Type"; sequence "CAAGAG"

## get the relative scores
relScore(siteset)

## [1] 0.6652 0.6652 0.6141 0.6634 0.6141 0.6634

relScore(sitesetList)

## $MA0003.2
## [1] 0.6197
##
## $MA0004.1
## [1] 0.6652 0.6652 0.6141 0.6634 0.6141 0.6634

## calculate the empirical p-values of the scores
pvalues(siteset, type="TFMPvalue")

## [1] 0.02734 0.02734 0.04639 0.04053 0.04639 0.04053

pvalues(siteset, type="sampling")

## [1] 0.0287 0.0287 0.0574 0.0401 0.0574 0.0401
```

6.2 searchAln

`searchAln` scans a pairwise alignment with the pattern represented by the PWM. It reports only those hits that are present in equivalent positions of both sequences and exceed a specified threshold score in both, AND are found in regions of the alignment above the specified.

```
library(Biostrings)
data(MA0003.2)
pwm <- toPWM(MA0003.2)
aln1 <- DNAString("ACTTCACCAGCTCCCTGGCGGTAAGTTGATC---AAAGG---AAACGCAAAGTTTTCAAG")
aln2 <- DNAString("GTTTCACTACTTCCTTTCGGGTAAGTAAATATATAAATATATAAAAATATAATTTTCATC")
sitePairSet <- searchAln(pwm, aln1, aln2, seqname1="seq1", seqname2="seq2",
                         min.score="50%", cutoff=0.5,
                         strand="*", type="any")
## generate gff style output
head(writeGFF3(sitePairSet))

##   seqname source feature start end   score strand frame
## 1    seq1   TFBS    TFBS     6  20  -9.515      +     .
## 2    seq1   TFBS    TFBS     7  21 -13.349      +     .
## 3    seq1   TFBS    TFBS     8  22 -13.182      +     .
## 4    seq1   TFBS    TFBS     9  23  -3.730      +     .
## 5    seq1   TFBS    TFBS    10  24  -7.678      +     .
## 6    seq1   TFBS    TFBS    14  28 -20.775      +     .
##                                                     attributes
```

```
## 1 TF=TFAP2A;class=Zipper-Type;sequence=ACCAGCTCCCTGGCG
## 2 TF=TFAP2A;class=Zipper-Type;sequence=CCAGCTCCCTGGCGG
## 3 TF=TFAP2A;class=Zipper-Type;sequence=CAGCTCCCTGGCGGT
## 4 TF=TFAP2A;class=Zipper-Type;sequence=AGCTCCCTGGCGGTA
## 5 TF=TFAP2A;class=Zipper-Type;sequence=GCTCCCTGGCGGTAA
## 6 TF=TFAP2A;class=Zipper-Type;sequence=CCTGGCGGTAAGTTG
```

```
head(writeGFF2(sitePairSet))
```

```
##   seqname source feature start end   score strand frame
## 1    seq1   TFBS    TFBS     6  20  -9.515      +     .
## 2    seq1   TFBS    TFBS     7  21 -13.349      +     .
## 3    seq1   TFBS    TFBS     8  22 -13.182      +     .
## 4    seq1   TFBS    TFBS     9  23  -3.730      +     .
## 5    seq1   TFBS    TFBS    10  24  -7.678      +     .
## 6    seq1   TFBS    TFBS    14  28 -20.775      +     .
##                                                  attributes
## 1 TF "TFAP2A"; class "Zipper-Type"; sequence "ACCAGCTCCCTGGCG"
## 2 TF "TFAP2A"; class "Zipper-Type"; sequence "CCAGCTCCCTGGCGG"
## 3 TF "TFAP2A"; class "Zipper-Type"; sequence "CAGCTCCCTGGCGGT"
## 4 TF "TFAP2A"; class "Zipper-Type"; sequence "AGCTCCCTGGCGGTA"
## 5 TF "TFAP2A"; class "Zipper-Type"; sequence "GCTCCCTGGCGGTAA"
## 6 TF "TFAP2A"; class "Zipper-Type"; sequence "CCTGGCGGTAAGTTG"
```

```
## search the Axt alignment
library(CNEr)
```

```
##
## Attaching package:  'CNEr'
##
## The following object is masked from 'package:Biostrings':
##
##    N50
```

```
axtFilesHg19DanRer7 <- file.path(system.file("extdata", package="CNEr"),
                                 "hg19.danRer7.net.axt")
axtHg19DanRer7 <- readAxt(axtFilesHg19DanRer7)
```

```
## The number of axt files 1
## The number of axt alignments is 133
```

```
sitePairSet <-  searchAln(pwm, axtHg19DanRer7, min.score="80%",
                          windowSize=51L, cutoff=0.7, strand="*",
                          type="any", conservation=NULL, mc.cores=2)
GRangesTFBS <- toGRangesList(sitePairSet, axtHg19DanRer7)
GRangesTFBS$targetTFBS
```

```
## GRanges object with 33 ranges and 5 metadata columns:
##        seqnames               ranges strand  |   matrix.ID matrix.strand abs.score
##           <Rle>            <IRanges>  <Rle>  | <character>   <character> <numeric>
##    [1]    chr11 [31128026, 31128040]      +  |    MA0003.2             +     2.089
##    [2]    chr11 [31128028, 31128042]      +  |    MA0003.2             -     1.405
##    [3]    chr11 [31437161, 31437175]      +  |    MA0003.2             +     7.786
##    [4]    chr11 [31437163, 31437177]      +  |    MA0003.2             -     7.797
##    [5]    chr11 [31499628, 31499642]      +  |    MA0003.2             +     2.239
```

```
##   ...      ...                  ...    ... ...          ...           ...       ...
##   [29]     chr11 [32198154, 32198168]      +   |    MA0003.2             +     2.512
##   [30]     chr11 [32221916, 32221930]      +   |    MA0003.2             +     4.672
##   [31]     chr11 [32221918, 32221932]      +   |    MA0003.2             -    10.610
##   [32]     chr11 [32456353, 32456367]      +   |    MA0003.2             +     4.205
##   [33]     chr11 [32456355, 32456369]      +   |    MA0003.2             -     1.177
##        rel.score        sitesSeq
##        <numeric>  <DNAStringSet>
##    [1]    0.8130 GGCCCCCCGTGGCGT
##    [2]    0.8059 CCCCCCGTGGCGTTT
##    [3]    0.8725 ACTGGCCTAGGGACA
##    [4]    0.8726 TGGCCTAGGGACAGG
##    [5]    0.8146 TCTGACCCAGAGGCA
##    ...       ...             ...
##   [29]    0.8174 ACCGTCTTTAGGGGA
##   [30]    0.8400 TCTACCCTCGAGCAC
##   [31]    0.9020 TACCCTCGAGCACTG
##   [32]    0.8351 AAGGGCCCGTAGCGA
##   [33]    0.8035 GGGCCCGTAGCGACA
##   -------
##   seqinfo: 3 sequences from an unspecified genome; no seqlengths
```

```
GRangesTFBS$queryTFBS
```

```
## GRanges object with 33 ranges and 5 metadata columns:
##        seqnames               ranges strand   |   matrix.ID matrix.strand abs.score
##           <Rle>            <IRanges>  <Rle>   | <character>   <character> <numeric>
##    [1]    chr25 [15057430, 15057444]      +   |    MA0003.2             +     2.657
##    [2]    chr25 [15057432, 15057446]      +   |    MA0003.2             -     5.006
##    [3]    chr25 [15149515, 15149529]      +   |    MA0003.2             +     5.686
##    [4]    chr25 [15149517, 15149531]      +   |    MA0003.2             -     6.435
##    [5]    chr25 [15163682, 15163696]      +   |    MA0003.2             +     1.493
##   ...      ...                  ...    ... ...          ...           ...       ...
##   [29]    chr25 [15359169, 15359183]      +   |    MA0003.2             +     3.135
##   [30]    chr25 [15472022, 15472036]      +   |    MA0003.2             +     1.341
##   [31]    chr25 [15472024, 15472038]      +   |    MA0003.2             -     5.674
##   [32]    chr25 [15570786, 15570800]      +   |    MA0003.2             +     1.605
##   [33]    chr25 [15570789, 15570803]      +   |    MA0003.2             -     5.262
##        rel.score        sitesSeq
##        <numeric>  <DNAStringSet>
##    [1]    0.8190 GGCCCCCTGCGGCTC
##    [2]    0.8435 CCCCCTGCGGCTCTC
##    [3]    0.8506 TCTCGCCTAAGGAAA
##    [4]    0.8584 TCGCCTAAGGAAAAA
##    [5]    0.8068 TGCCACCCTTGGGCA
##    ...       ...             ...
##   [29]    0.8239 ACCATCTTTAGGGGA
##   [30]    0.8052 TCTCCCCTCCAGCTC
##   [31]    0.8504 TCCCCTCCAGCTCTG
##   [32]    0.8080 CCGTACCTGCAGGCC
##   [33]    0.8461 TACCTGCAGGCCCCT
##   -------
```

```
##   seqinfo: 3 sequences from an unspecified genome; no seqlengths
```

6.3 searchPairBSgenome

`searchPairBSgenome` is designed to do the genome-wise phylogenetic footprinting. Given two *BSgenome*, a chain file for liftover from one genome to another, `searchPairBSgenome` identifies the putative transcription factor binding sites which are conserved in both genomes.

```
library(rtracklayer)
library(JASPAR2014)
library(BSgenome.Hsapiens.UCSC.hg19)
library(BSgenome.Mmusculus.UCSC.mm10)
pfm <- getMatrixByID(JASPAR2014, ID="MA0004.1")
pwm <- toPWM(pfm)
chain <- import.chain("Downloads/hg19ToMm10.over.chain")
sitePairSet <- searchPairBSgenome(pwm, BSgenome.Hsapiens.UCSC.hg19,
                                  BSgenome.Mmusculus.UCSC.mm10,
                                  chr1="chr1", chr2="chr1",
                                  min.score="90%", strand="+", chain=chain)
```

7 Use de novo motif discovery software

In this section, we will introduce wrapper functions for external motif discovery programs. So far, *MEME* is supported.

7.1 MEME

`runMEME` takes a *DNAStringSet* or a set of *characters* as input, and returns a *MotifSet* object.

```
motifSet <- runMEME(file.path(system.file("extdata",
                                          package="TFBSTools"), "crp0.s"),
                    binary="meme",
                    arguments=list("-nmotifs"=3)
                   )
## Get the sites sequences and surrounding sequences
sitesSeq(motifSet, type="all")
## Get the sites sequences only
sitesSeq(motifSet, type="none")
consensusMatrix(motifSet)
```

8 Session info

The following is the session info that generated this vignette:

```
  sessionInfo()

## R Under development (unstable) (2015-10-18 r69537)
## Platform: x86_64-apple-darwin15.0.0 (64-bit)
```

```
## Running under: OS X 10.11.1 (El Capitan)
##
## locale:
## [1] en_US.UTF-8/en_US.UTF-8/en_US.UTF-8/C/en_US.UTF-8/en_US.UTF-8
##
## attached base packages:
## [1] stats4    parallel  stats     graphics  grDevices utils     datasets  methods
## [9] base
##
## other attached packages:
##  [1] CNEr_1.7.0           JASPAR2014_1.7.0     Biostrings_2.39.0    XVector_0.11.0
##  [5] IRanges_2.5.5        S4Vectors_0.9.6      BiocGenerics_0.17.0  TFBSTools_1.9.3
##  [9] knitr_1.11           BiocInstaller_1.21.1
##
## loaded via a namespace (and not attached):
##  [1] Rcpp_0.12.1                 formatR_1.2.1               futile.logger_1.4.1
##  [4] GenomeInfoDb_1.7.3          highr_0.5.1                 bitops_1.0-6
##  [7] futile.options_1.0.0        tools_3.3.0                 zlibbioc_1.17.0
## [10] digest_0.6.8                RSQLite_1.0.0               evaluate_0.8
## [13] BSgenome_1.39.0             DBI_0.3.1                   DirichletMultinomial_1.13.0
## [16] seqLogo_1.37.0              rtracklayer_1.31.1          stringr_1.0.0
## [19] gtools_3.5.0                caTools_1.17.1              grid_3.3.0
## [22] Biobase_2.31.0              XML_3.98-1.3                BiocParallel_1.5.0
## [25] lambda.r_1.1.7              magrittr_1.5                Rsamtools_1.23.1
## [28] codetools_0.2-14            GenomicRanges_1.23.1        GenomicAlignments_1.7.2
## [31] SummarizedExperiment_1.1.1  BiocStyle_1.9.2             TFMPvalue_0.0.5
## [34] stringi_1.0-1               RCurl_1.95-4.7
```

References

[1] Boris Lenhard and Wyeth W Wasserman. TFBS: Computational framework for transcription factor binding site analysis. *Bioinformatics*, 18(8):1135–1136, August 2002.

[2] Anthony Mathelier and Wyeth W Wasserman. The next generation of transcription factor binding site prediction. *PLoS Comput. Biol.*, 9(9):e1003214, 2013. doi:10.1371/journal.pcbi.1003214.

[3] T L Bailey and C Elkan. Fitting a mixture model by expectation maximization to discover motifs in biopolymers. *Proc Int Conf Intell Syst Mol Biol*, 2:28–36, 1994. PMID: 7584402.

[4] Wyeth W Wasserman and Albin Sandelin. Applied bioinformatics for the identification of regulatory elements. *Nature Publishing Group*, 5(4):276–287, April 2004.

[5] Keishin Nishida, Martin C Frith, and Kenta Nakai. Pseudocounts for transcription factor binding sites. *Nucleic Acids Res.*, 37(3):939–944, February 2009. PMID: 19106141. doi:10.1093/nar/gkn1019.

[6] T D Schneider, G D Stormo, L Gold, and A Ehrenfeucht. Information content of binding sites on nucleotide sequences. *J. Mol. Biol.*, 188(3):415–431, April 1986. PMID: 3525846.

[7] Albin Sandelin, Annette Hglund, Boris Lenhard, and Wyeth W Wasserman. Integrated analysis of yeast regulatory sequences for biologically linked clusters of genes. *Funct. Integr. Genomics*, 3(3):125–134, July 2003. doi:10.1007/s10142-003-0086-6.

[8] Chaim Linhart, Yonit Halperin, and Ron Shamir. Transcription factor and microRNA motif discovery: the amadeus platform and a compendium of metazoan target sets. *Genome Res.*, 18(7):1180–1189, July 2008. PMID: 18411406. doi:10.1101/gr.076117.108.

[9] Jan Christian Bryne, Eivind Valen, Man-Hung Eric Tang, Troels Marstrand, Ole Winther, Isabelle da Piedade, Anders Krogh, Boris Lenhard, and Albin Sandelin. JASPAR, the open access database of transcription factor-binding profiles: new content and tools in the 2008 update. *Nucleic Acids Res.*, 36(Database issue):D102–106, January 2008. doi:10.1093/nar/gkm955.
